# Blueberry muffin syndrome

**Published:** 2012-10-02

**Authors:** Sarra Benmiloud, Ghizlane Elhaddou, Zoubida Alaoui Belghiti, Moustapha Hida, Abdelhak Bouharrou

**Affiliations:** 1 Unité d'hémato-oncologie pédiatrique, service de pédiatrie, CHU Hassan II, Fès, Maroc; 2 Service de néonatologie et réanimation néonatale, CHU Hassan II, Fès, Maroc

**Keywords:** Blueberry Muffin Baby, Leucémie myéloïde, Nouveau-né, Hématopoï èse dermique

## Abstract

Le Blueberry Muffin Baby est un syndrome cutané rare observé en période néonatale. Il est caractérisé par des papulo-nodules disséminés inflammatoires traduisant des réactions d'hématopoïèse dermique. Plusieurs causes doivent être recherchées, notamment les infections congénitales, une hémolyse sévère et les pathologies tumorales. Nous rapportons l'observation d'un nouveau-né chez qui l'aspect d'un Blueberry muffin baby a conduit au diagnostic d'une leucémie aiguë myéloïde.

## Introduction

Le Blueberry Muffin Baby est un syndrome cutané rare observé en période néonatale. Il est caractérisé par des papulo-nodules disséminés inflammatoires allant du rouge vif au bleu gris traduisant des réactions d'hématopoïèse dermique [1, 2]. Plusieurs causes doivent être recherchées, notamment les infections congénitales, les maladies hémolytiques néo-natales et les pathologies tumorales [2, 3]. Nous rapportons l'observation d'un nouveau-né chez lequel l'aspect d'un Blueberry muffin baby a conduit au diagnostic d'une leucémie aiguë myéloïde.

## Patient et observation

Nouveau-né de sexe masculin, de phénotype normal, sans notion de consanguinité des parents ni d'antécédent particulier, issu d'une grossesse menée à terme, suivie avec une anamnèse infectieuse négative, l'accouchement était par voie basse avec une bonne adaptation à la vie extra-utérine. Il est admis le jour de sa naissance au service de réanimation néonatale pour prise en charge d'une détresse respiratoire associée à une éruption cutanée généralisée.

**Figure 1:**
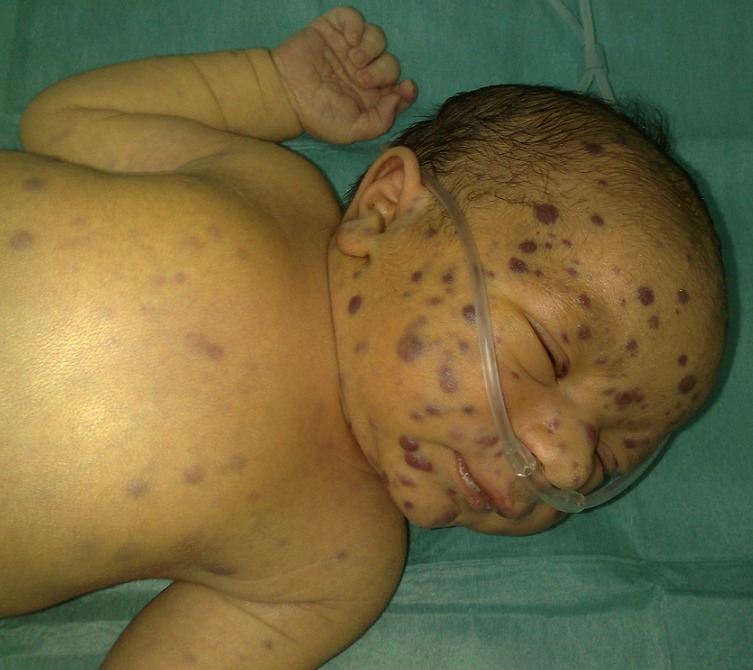
Lésions papulo-nodulaires érythémato-bleutées typiques d'un du Blueberry muffin syndrome

L'examen clinique à l'admission met en évidence plusieurs lésions cutanées maculo-papuleuses et nodulaires érythémato-bleutées et violacées diffuses sur tout le corps, de taille variant entre 4 et 8 mm de diamètre, prédominant au niveau du visage et du cuir chevelu, réalisant un tableau de Blueberry muffin baby (Figures 1 et 2), une hépatomégalie à 3 travers de doigts par rapport au rebord costal droit, une splénomégalie à 2 travers de doigts par rapport au rebord costal gauche, une détresse respiratoire avec un score de silverman à 5/10 sans râles à l'auscultation pulmonaire, et un testicule droit augmenté de volume et infiltré.

**Figure 2:**
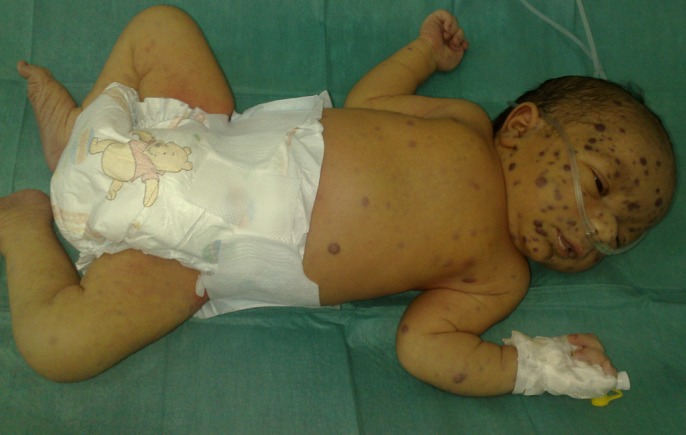
Lésions du Blueberry muffin syndrome disséminées au niveau de tout le corps prédominant au niveau de la tête, du cou et du tronc

Le bilan biologique a mis en évidence une hyperleucocytose à 88000/mm3 (polynucléaires neutrophiles = 37%, lymphocytes = 57%, monocytes = 5%) avec présence de 33% de blastes au frottis sanguin, une thrombopénie à 66000/mm3 et une anémie à 10 g/dl. Les LDH sont à 4840 UI/L et l'acide urique à 93 mg/l. Le bilan infectieux a montré une CRP à 125 mg/l avec hémoculture positive à *Entercoccus faecalis*. Les sérologies TORSCH (toxoplasmose, rubéole, syphilis, cytomégalovirus, herpes) sont négatives aussi bien chez le bébé que chez sa maman. Le nouveau-né est de groupe A rhésus positif et la maman est de groupe O rhésus positif. Le bilan d'hémolyse est normal. Le myélogramme a montré une infiltration par 38% de blastes indifférenciés et de myéloblastes à noyaux irrégulier, à chromatine finement réticulée avec un cytoplasme basophile granulé. L’étude immuno-phénotypique a montré qu'ils sont positifs pour des leucocytes humains antigens (HLA-DR), CD13, CD14, CD33 et CD68 en faveur d'une leucémie aigue myéloïde (LAM) de type monoblastique (LAM 5 selon la classification franco-américano-britannique «FAB»). L’étude cytogénétique n'a pu être réalisée par manque de moyens financiers chez les parents. La radiographie thoracique n'a pas montré d’élargissement médiastinal ni de signes de leucostase. L’échographie abdominale a montré une hépato-splénomégalie homogène.

Le nouveau-né est mis sous oxygénothérapie (3l/min), une bi-antibiothérapie (ceftriaxone 100 mg/kg/j + gentamycine 5 mg/kg/j), une hyperhydratation alcaline et l'allopurinol. L’évolution est marquée par une aggravation de l'hyperleucocytose qui a atteint 200000/mm3 avec 85% de blastes et une aggravation de l’état infectieux malgré l'escalade de l'antibiothérapie (pénicilline A + ceftazidime + amikacine). Le nouveau-né est décédé 13 jours plus tard dans un tableau de septicémie.

## Discussion

Le syndrome Blueberry Muffin Baby correspond à des papulo-nodules disséminés, présents dés la naissance et caractérisés par des éléments allant du rouge vif au bleu gris [1, 2]. Il s'agit d'une éruption souvent généralisée prédominant au niveau de la tête, du cou et du tronc, mesurant environ 2 à 8 mm de diamètre [2, 4]. Ces lésions disparaissent généralement au bout de 3 à 6 semaines après la naissance en prenant progressivement une coloration marron pâle. Ce syndrome représente une expression post-natale de l'hématopoïèse dermique qui peut persister après la naissance si le stress érythropoïétique est sévère ou bien il peut correspondre à une infiltration néoplasique [4, 5].

En période néonatale, l'hématopoïèse dermique est liée aux infections congénitales telles que la toxoplasmose, la rubéole, le cytomégalovirus, le virus coxackie B2 et le parvovirus B19, ou bien à une hémolyse sévère en rapport avec une incompatibilité rhésus ou une incompatibilité dans le système ABO, la sphérocytose héréditaire, ou bien le syndrome transfuseur-transfusé chez les jumeaux [2, 6]. Parmi les affections malignes associées à un bleuberry muffin syndrome, le neuroblastome est l'affection la plus fréquente, alors que le rhabdomyosrcome, l'histiocytose et la leucémie congénitale ou néonatale sont très rares [2, 3, 7, 8]. Les critères de malignité d'un nodule cutané sont le caractère explosif des lésions cutanées, l'altération de l’état général, les adénopathies, l'hépato-splénomégalie, l'aspect induré et la coloration bleutée [1].

Le diagnostic du Blueberry Muffin syndrome a été aisé chez notre patient qui présentait des lésions cutanées typiques. La conduite du diagnostic étiologique était facile car on avait une hyperleucocytose importante, chez un nouveau-né de morphotype normal, associée à une blastose sanguine et médullaire avec absence d'infection congénitale ou incompatibilité materno-fœtale. Le diagnostic de leucémie néonatale de type LAM 5 a été objectivé par le médullogramme et l'immuno-phénotypage.

Malgré sa rareté, une leucémie congénitale est un des premiers diagnostics à évoquer devant un tableau de Blueberry Muffin baby. Il s'agit le plus souvent d'une leucémie de type myéloïde, et notamment les LAM 4 et LAM 5 [2, 9]. La présence de leucémides dans un contexte de leucémie congénitale n'est pas un facteur de mauvais pronostic mais le pronostic global de ces leucémies est pauvre, en particulier en cas de mutation (t11-19) du gène MLL (mixed-lineage-leukemia). Avant de porter le diagnostic de leucémie congénitale, il faut s'assurer de l'absence d'une pathologie pouvant entraîner une réaction «leucémoïde» (notamment l'incompatibilité fœto-maternelle et les infections intra-utérines, par persistance d'une hématopoïèse cutanée) et d'une pathologie associée à une «hématopoïèse instable», comme par exemple, la trisomie 21 et le syndrome de Noonan [10]. Dans notre cas, l’étude cytogénétique n'a pu être réalisée par manque de moyens financiers chez les parents, chose qui ne nous a pas permis d’éliminer catégoriquement une anomalie chromosomique constitutionnelle responsable d'une hématopoïèse instable malgré le morphotype normal du nouveau-né.

## Conclusion

Devant tout Blueberry Muffin Baby, il paraît important d’évoquer une leucémie congénitale malgré sa rareté à cet âge. Cependant, il peut s'agir de réactions d'hématopoïèse dermique en rapport avec des infections congénitales ou des maladies hémolytiques, ou bien des métastases de neuroblastome, de rhabdomyosarcome, voire une histiocytose.
